# CD38 inhibition increases neuronal survival and reduces α-synuclein *in vitro*, elevates brain NAD^+^, and reduces motor deficits in a Parkinson’s disease model

**DOI:** 10.3389/fphar.2026.1927552

**Published:** 2026-10-23

**Authors:** Jenna R. M. Harvey, Thomas H. Wight, Maxine Roberts, Victoria Mulligan, Daniel F. Barker, Chloe Sherlock, Bernardino Ossola, Richard Hewer, Xiao Xu, Keith Page, Justin Powell, Roland W. Bürli, Nigel Ramsden, Lee A. Dawson, Mark B. Carlton, Nicola L. Brice, Andrew J. Stott

**Affiliations:** Cerevance Ltd, Cambridge, United Kingdom

**Keywords:** AAV1/2-hA53T, α-synuclein, CD38, NAD^+^, neurodegeneration, Parkinson's disease

## Abstract

**Introduction:**

Nicotinamide adenine dinucleotide (NAD^+^), an essential cofactor for multiple cellular processes, is critical for cellular health. NAD^+^ declines with age due to reduced biosynthesis and increased consumption. This causes cellular dysfunction, which exacerbates and contributes to the pathology of age-related CNS diseases, including Parkinson’s disease (PD). CD38 is an NAD^+^-consuming enzyme (NADase) that increases in expression with ageing and disease, likely contributing to the age-related decline in NAD^+^. Genetic links between CD38 and PD provide a rationale for exploring its function in disease.

**Methods:**

NETSseq datasets from human post-mortem brain tissue were used to assess CD38 cell-type-specific expression. The pharmacological properties of CVN14, a brain-penetrant selective inhibitor previously described by our group, were characterised using *in vitro* CD38 enzyme assays, selectivity profiling, pharmacokinetic and brain penetration studies, and brain NAD^+^ measurements in rats and mice. The effects of CD38 inhibition were evaluated in α-synuclein-challenged rat mesencephalic cultures and in a rat AAV1/2-hA53T α-synuclein model of PD.

**Results:**

CD38 was highly enriched in astrocytes in the human brain. CVN14 potently inhibited recombinant human, rat, and mouse CD38, with IC_50_ values of 19.0, 5.1, and 2.4 nM, respectively. This compound was selective, having no activity at its closest homologue, CD157, or at other NADases. CVN14 increased brain NAD^+^ in rats and mice with no effect in the CD38 knockout mouse, further demonstrating its selectivity against other NADases. In a rat mesencephalic culture, CVN14 showed evidence of increasing dopaminergic neuronal survival following α-synuclein-induced toxicity and of reducing the number of neurons positive for pSer129 α-synuclein, thus supporting further investigation into a potential role for CD38 inhibition in the clearance of pathological protein. In a rat AAV1/2-hA53T α-synuclein model of PD, CVN14 improved motor function in the cylinder test.

**Conclusion:**

Our findings support further the investigation of CD38 inhibition as a therapeutic approach for PD. CVN14 elevated brain NAD^+^ in rats and mice reduced motor deficits in a rat α-synuclein model of PD and was associated with increased neuronal survival and reduced pathological α-synuclein *in vitro*, highlighting the therapeutic potential of CD38 inhibition in PD and other α-synucleinopathies.

## 1 Introduction

Cluster of differentiation 38 (CD38) is a multifunctional enzyme that plays a central role in maintaining cellular and tissue nicotinamide adenine dinucleotide (NAD^+^) homeostasis, primarily by catalysing the hydrolysis of NAD^+^ to nicotinamide and adenosine diphosphate ribose (ADPR). NAD^+^ is an essential cellular cofactor in most living cells and is critical for many cellular redox functions by serving as a primary electron carrier. NAD^+^ and its reduced form, NADH, play an integral part in oxidative phosphorylation and cellular bioenergetics in a broader context. However, the biological relevance of NAD^+^ extends beyond its role as a metabolic cofactor. NAD^+^ and its intermediates act as substrates for multiple enzyme families, including sirtuins (Hall et al., 2013), sterile alpha and TIR motif-containing protein 1 (SARM1) (Essuman et al., 2017), and poly (ADP-ribose) polymerases (PARPs) (Murata et al., 2019). This couples NAD^+^ to diverse cellular signalling pathways and thus to a central role in regulating homeostasis.

CD38 functions as the dominant NAD^+^-metabolising enzyme (NADase) in the brain, thereby controlling the bioavailability of NAD^+^ (Chini, 2009). The products of the CD38 consumption of NAD^+^, ADPR and cyclic-ADPR (cADPR), act as second messengers involved in intracellular calcium signalling (Zeidler et al., 2022). However, CD38 is a relatively inefficient generator of intracellular second messengers and is predominantly orientated in the plasma membrane, with its catalytic region exposed to the extracellular space (Chini, 2009; Malavasi et al., 2008). Consequently, its principal biological role is likely the regulation of NAD^+^ homeostasis, as reflected by its high substrate affinity and rapid turnover compared with other NADases (Zeidler et al., 2022).

It is well established that cellular NAD^+^ levels reduce during chronological ageing, leading to metabolic dysfunction and disease (Chini et al., 2017; Imai and Guarente, 2014; Schultz and Sinclair, 2016; Verdin, 2015). Parkinson’s disease (PD) is one such age-associated neurodegenerative disorder where enhancing NAD^+^ may correct metabolic deficits (Chini et al., 2017), as evidenced by studies demonstrating that increasing NAD^+^ improved health and lifespan in humans and in animal models of ageing and neurodegenerative disease (Covarrubias et al., 2021; Fang et al., 2016; Lautrup et al., 2019; Lehmann et al., 2017; Martens et al., 2018; Mitchell et al., 2018; Pérez et al., 2021). Consequently, interest has grown in progressing both pharmacological and nutraceutical therapeutics that boost NAD^+^.

Since NAD^+^ concentrations are significantly higher in mice that are genetically deficient in CD38 compared to their wild-type counterparts, CD38 may be the most relevant NADase for controlling NAD^+^ levels in mammals (Camacho-Pereira et al., 2016; Tarragó et al., 2018; Young et al., 2006). CD38 expression and activity increase with age and are required for mitochondrial dysfunction associated with age-related NAD^+^ decline (Camacho-Pereira et al., 2016). In addition, reduced NAD^+^ levels have been found across various neurodegenerative diseases such as PD (Wakade et al., 2014), Alzheimer’s disease (AD) (Sonntag et al., 2017), amyotrophic lateral sclerosis (Wang et al., 2017), and multiple sclerosis (Braidy et al., 2013). Inhibiting CD38 therefore presents a feasible way to therapeutically increase cellular NAD^+^ levels, thus reversing the dysfunction of mechanisms that contribute to neurodegenerative disease.

Knocking out CD38 in mice substantiates this hypothesis, leading to beneficial effects in neurodegenerative disease models (Blacher et al., 2015; Long et al., 2017; Takaso et al., 2020) and neuroinflammatory disease (Choe et al., 2011; Roboon et al., 2021). Furthermore, a non-brain-penetrant CD38 inhibitor, 78c, administered to mice has mitigated the age-related reduction in tissue NAD^+^ and the physiological effects of ageing (Tarragó et al., 2018). Crossing CD38 KO mice with the AD mouse model, APPswePS1DE9, reduced amyloid plaque load and improved performance in a learning and memory task (Blacher et al., 2015). In stroke models, CD38 KO mice had decreased expression of proinflammatory cytokines, reduced ischaemic injury, and reduced neurological deficits (Choe et al., 2011; Long et al., 2017). In mouse models of multiple sclerosis, CD38 KO improved outcomes and reduced neuroinflammation in experimental autoimmune encephalomyelitis and a model of demyelination, respectively (Herrmann et al., 2016; Roboon et al., 2019). In addition, CD38 KO or NAD^+^ administration was protective in a mouse facial nerve axotomy model (Takaso et al., 2020). This body of biological evidence supporting the therapeutic investigation of CD38 inhibition sits alongside human genetic studies that propose CD38 as a PD risk-associated gene. Several genetic and proteomic analyses suggest that genetically predicted lower CD38 expression or protein abundance is associated with increased PD risk, whereas others primarily support a role for CD38 without clearly resolving the directionality (Ge et al., 2023; Gu et al., 2023; Kia et al., 2021; Shi et al., 2024; Yao et al., 2021; Zhou et al., 2023). Together, these findings provide a rationale for evaluating pharmacological CD38 inhibition in disease-relevant models of PD. While CD38 is recognised as an important regulator of age-associated NAD^+^ decline, this study was designed to determine whether CD38 inhibition can modify PD-relevant mechanisms and endpoints rather than to investigate the biology of ageing itself. Pharmacological investigation has largely been hampered by the absence of brain-penetrant CD38 inhibitors.

Here, we demonstrate significant preclinical evidence that supports the utility of augmenting cellular NAD^+^ concentration by reducing its breakdown via CD38 inhibition. We characterise a novel, brain-penetrant CD38 inhibitor, CVN14, and investigate its ability to protect dopaminergic neurons, reduce pathological α-synuclein accumulation *in vitro*, and alleviate α-synuclein-induced motor deficits in a rat model of PD.

## 2 Materials and methods

### 2.1 Compounds and reagents

CVN14 was generated at WuXi AppTec, Shanghai, China (Figure 1). Synthetic methods are outlined in Stott et al., 2026. For *in vivo* dosing, CVN14 was formulated in 0.5% methylcellulose/0.2% Tween 80 in distilled water.

**FIGURE 1:**
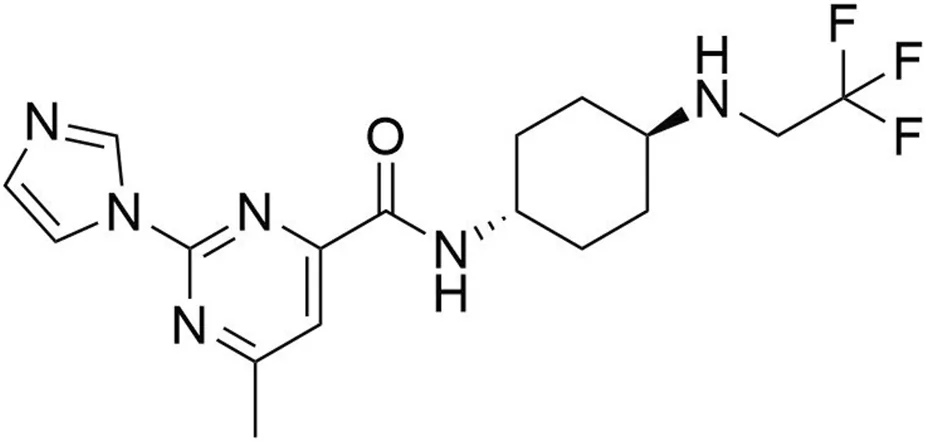
Structure of CVN14.

### 2.2 Human tissue

Human brain samples were procured from the following sources: Douglas-Bell Canada Brain Bank (Douglas Bell Hospital Research Centre, Montreal, Quebec, Canada), Maryland Brain Bank (University of Maryland, Baltimore, United States), Miami Brain Endowment Bank and NIH NeuroBioBank (University of Miami, Miami, United States), Netherlands Brain Bank (Amsterdam, Netherlands), South West Dementia Brain Bank (Southmead Hospital, Bristol, United Kingdom), and Tissue4Research (Newmarket, United Kingdom). All human specimens analysed in this investigation were obtained from donors who had provided full written consent permitting the use of their biological materials and associated clinical data for research purposes. The procurement and use of these samples adhered to the World Medical Association’s Declaration of Helsinki. Handling, storage, and governance of both data and tissue were conducted in accordance with the requirements of the United Kingdom *Human Tissue Act*. We sincerely thank all participating brain banks for generously providing brain tissue for this study, and the donors and their families for making this research possible. Nuclear Enriched Transcript Sort sequencing (NETSseq) data include control donors (n = 230; 126 female and 104 male donors) who were not diagnosed with a central nervous system (CNS)-related disorder and whose causes of death were unrelated to the CNS. The median age of donors was 78.5 (range, 8–104) years with a mean post-mortem delay of 30.0 (range, 3.0–153.0) hours.

### 2.3 Nuclear Enriched transcript sort sequencing (NETSseq) methods

Isolated nuclei were prepared from human brain samples as per Stirparo et al. (2025) and Xu et al. (2018). In brief, human CNS tissue samples were homogenised, and nuclei were isolated using an iodixanol gradient. Resuspended nuclei were fixed with 1% formaldehyde and labelled with NeuN primary antibody (1:250, Millipore, MAB377) and SLC1A3 fluorescently conjugated antibody (1:125, Thermo Fisher Scientific, PA5-72895), followed by labelling with Alexa Fluor 488/594 secondary antibodies (1:250, Jackson ImmunoResearch, 715–545–151/715–585–151). Nuclei were co-stained with DAPI (0.01 mg/mL) and then sorted by flow cytometry (BD FACSAria Fusion; BD Biosciences, San Jose, CA, United States) using 355 nm, 488 nm, 561 nm, and 640 nm lasers. Forward and side scatter gates excluded debris, and a single nuclei DAPI gate excluded aggregates. Cell types of interest were isolated using gates based on antibody labelling. FlowJo and FACSDiva (BD) software were used for analysis. Nuclei were sorted at 4 °C and stored at −80 °C prior to RNA extraction.

### 2.4 Histology

CD38 expression was evaluated in human brain sections using 4 µm formalin-fixed, paraffin-embedded tissue and fully automated, chromogenic immunohistochemical methods (Agilent Envision FLEX plus). Monoclonal antibodies against human CD38 were acquired from Cell Signaling Technology (#51000, rabbit anti-human CD38, clone E7Z8C, 1/20) and validated internally using spleen and overlapping immunofluorescence with glial fibrillary acidic protein (GFAP) in astrocytes (Supplementary Figure S1). GFAP antibody (clone GA5 mlG1) was used at a concentration of 1:2000 and was detected using anti-mouse DyLight 594 fluorophore at 1:200 (10 mg/mL). CD38 mRNA was visualised in frozen sections of human brain (10 µm) using RNAscope 2.5 HD Assay Red and RNAscope 2.5 HD Duplex Assay (ACD Biosystems, Bio-Techne).

### 2.5 Enzyme activity

A fluorescence-based assay using a non-physiological NAD^+^ substrate analogue (1,N6-etheno NAD^+^: ε-NAD (Sigma-Aldrich)) was used to assess CVN14 inhibition of CD38 enzymatic activity. CVN14 was incubated with recombinant CD38 (BPS Bioscience) (human 0.8 nM; rat: 0.8 nM; mouse: 0.4 nM) in phosphate-buffered saline (PBS) (-Ca^2+^/Mg^2+^) containing 0.005% bovine serum albumin (BSA) (pH 7.4) at 25 °C for 30 min in 384-well black microplates. CD38 activity was initiated by the addition of ε-NAD at K_m_ concentration (hCD38: 4 μM; rCD38: 14 μM; mouse: 12 µM), which yields the fluorescent product 1,N6-etheno ADP-ribose. The formation of fluorescent product was followed using a CLARIOstar Plus (BMG) microplate reader by reading fluorescence (excitation λ = 300 nm; emission λ = 410 nm) at two time points: one immediately after substrate addition (t = 0) and one during the linear phase of reaction at 10 min (t = 10, hCD38), 7 min (t = 7, rCD38), or 6 min (t = 6, mCD38). Data were analysed by subtracting the values detected at t = 0 from t = 10, 7, or 6 to correct for baseline fluorescence variation. Fluorescence values were converted to percentage inhibition using the average of high signal (CD38 and ε-NAD) and low signal (CD38 and ε-NAD in the presence of a tool CD38 inhibitor) control wells. IC_50_ values were determined from a ten-point, half log concentration response curve with a four-parameter logistic equation. The inhibition of cluster of differentiation 157 (CD157) activity was assessed following the above protocol using recombinant human CD157 (120 nM) and NAD^+^ substrate analogue nicotinamide guanine dinucleotide (NGD; 85 µM) in 50 mM MES, 2.5 mM ZnCl_2_ + 0.005% BSA, pH 6.5. Fluorescence readings were taken at 0 and 60 min.

### 2.6 Animals

All animal experimental procedures were performed in compliance with local guidelines and approved by local ethical review boards in the United Kingdom, China, and Canada. CD38 KO mice were generated at The Jackson Laboratory (Bar Harbor, Maine, United States) from B6.129P2-Cd38tm1Lnd/J (Stock #003727). Male KO mice aged 6–7 weeks and age-matched C57BL/6J males (Stock #000664) as wild-type (WT) controls were used for the NAD^+^ study.

### 2.7 *Ex vivo* brain NAD^+^

Adult Sprague–Dawley rats (female, 160–194 g), vehicle: n = 5, 1 mg/kg: n = 5, 3 mg/kg: n = 3, 10 mg/kg: n = 5, were administered CVN14 or vehicle *p.o.* Similarly, 6–7-week-old CD38 KO and WT mice, n = 5 per group, were administered 10 mg/kg CVN14 or vehicle *p.o.* Two hours after dosing, blood was collected by cardiac puncture under terminal anaesthesia with isofluorine. Their brains were harvested following cervical dislocation. The cerebellum was removed for bioanalysis, and the remaining brain was hemisected, snap-frozen, and stored at −80 °C. Brain hemispheres were homogenised in lysis buffer (20% DTAB in 0.2 M NaOH) at 10 μL per mg of tissue using an electric tissue homogeniser. Samples were centrifuged at 8,000 *× g* for 5 min, the supernatant removed and centrifuged again at 16,000 g for 15 min, before dilution for the assay. Brain NAD^+^ concentrations were quantified using the NAD/NADH-Glo assay kit (Promega) as per the manufacturer’s recommendations. Samples were treated under conditions that selectively measured the oxidised NAD^+^ pool while eliminating or preventing the detection of NADH with the addition of HCl.

### 2.8 Pharmacokinetic (PK) analysis

To determine PK properties of CVN14, female Sprague–Dawley rats (n = 3) were administered 3 mg/kg *p.o.* via gavage in a 1 mg/mL suspension. Blood (250 μL) was collected at 0.25, 0.5, 1, 2, 4, 8, and 24 h in serial bleeds from three rats into EDTA-K2 tubes. Blood samples were processed to plasma by centrifugation, frozen on dry ice, and stored at −60 °C until analysis by liquid chromatography-tandem mass spectrometry. Concentration versus time was plotted in Phoenix WinNonlin 6.3 and C_max_ (maximum concentration), and T_max_ (time at C_max_), T_last_ (time at last quantifiable concentration), and AUC_0–last_ (total exposure over the observed sampling period) were calculated. Free brain concentration at 8 h was determined from rats (n = 3) administered 10 mg/kg CVN14 and used to calculate unbound partition coefficient (Kp_u,u_). Free fraction was calculated from plasma–protein and brain-tissue binding assessments via *in vitro* equilibrium dialysis studies.

### 2.9 Dopaminergic neuronal survival and α-synuclein

Rat ventral mesencephalon cultures containing dopaminergic neurons and glia were prepared from the ventral portion of the mesencephalic flexure (where 2%–5% of neurons are dopaminergic) of E18 Wistar rats, as per Schinelli et al., 1988. α-Synuclein peptide (rPeptide, ref: S 1001–2, Batch: 070921AS) was reconstituted in culture medium at 4 μM and slowly shaken at 37 °C for 3 days in the dark. The control medium was prepared similarly. After 7 days of culture, cells were pre-treated for 2 h with CVN14, vehicle, or the positive controls: brain‐derived neurotrophic factor (BDNF; 50 ng/mL) or nicotinamide mononucleotide (NMN; 5 mM) before treatment with α-synuclein (500 nM) for 4 days. Cultures were generated from two independent preparations, each derived from pooled embryos from a separate litter. Six replicate wells were included per treatment condition within each preparation. For data presentation, replicate wells were summarised within each neuronal preparation, and the two independent preparations are shown as the biological replicates.

Cells were then fixed with 4% paraformaldehyde for 20 min at room temperature, permeabilised and blocked with PBS containing 0.1% saponin and 1% foetal bovine serum (FBS) for 15 min. Cells were incubated with a chicken polyclonal anti-tyrosine hydroxylase antibody (TH, 1/1,000, Abcam; ref: ab76442, Batch: GR3232849-2) and a mouse monoclonal anti-α-synuclein pSer129 antibody (1/500, Abcam; Ref: ab184674, Batch: GR3450549-2) in a solution of PBS overnight at 4 °C. Staining was determined using Alexa Fluor 568 goat anti-mouse IgG (1/400, Molecular Probes, ref: A11004, Batch: 2332536) and Alexa Fluor 647 goat anti-chicken IgG (1/400, Molecular Probes, ref: A21449, Batch: 2335730) in PBS with 1% FBS and 0.1% saponin for 1 h at room temperature. The nuclei of cells were labelled with a fluorescent marker (Hoechst 33,258, Sigma; ref: B1155, Batch: 046M4048V) in the same solution. For each condition, 20 images per well were taken at ×20 magnification using In Cell Analyzer™ 2200 (GE Healthcare). Analysis of TH-positive neuron cell bodies and pSer129 α-synuclein was performed using Developer software (GE Healthcare).

### 2.10 AAV1/2-hA53T-α-synuclein rat model of PD

AAV1/2-hA53T-α-synuclein or AAV1/2 empty vector (2 μl of 3 × 10^12^ genomic particles/mL) were administered unilaterally into the right substantia nigra (SN) (medial-lateral: 2.0 mm; antero-posterior: 5.2 mm; dorso-ventral: 7.5 mm) of female Sprague–Dawley rats randomised into five treatment groups (vehicle: n = 12, α-syn/vehicle: n = 14, α-syn/0.3 mg/kg: n = 11, α-syn/1 mg/kg: n = 13, α-syn/10 mg/kg: n = 12). see Koprich et al., 2011 for additional details. This model has been extensively validated in female rats, and thus females were used in the current study following confirmation of PK equivalency in females. One day after surgery, vehicle or CVN14 (0.3, 1, 10 mg/kg) was administered *p.o.* once daily, for 42 days. Behaviour was assessed using the cylinder test of forelimb asymmetry prior to surgery and on day 42. Animals were individually placed in a clear cylinder for 6 min, and the number of rears and contacts each forepaw made with the side of the cylinder was video-recorded and subsequently noted by an observer blinded to the treatments given. The first forepaw to contact the cylinder in every rear was given a score of “1”. If both limbs contacted within 0.4 s of each other, it was scored “both”. Animals with fewer than ten total contacts were excluded from the behavioural analysis because behavioural asymmetry measures are not considered reliable with insufficient exploratory activity.

On day 43, the rats were deeply anaesthetised with isoflurane, and blood samples were collected via cardiac puncture. They were then sacrificed by exsanguination via transcardial perfusion with ice-cold 0.9% saline containing 0.2% heparin, and the brain was extracted. Sections containing SN were prepared (animal numbers-vehicle: n = 7, α-syn/vehicle: n = 10, α-syn/0.3 mg/kg: n = 11, α-syn/1 mg/kg: n = 11, α-syn/10 mg/kg: n = 8), and pSer129 α-synuclein was detected immunohistochemically using ab51253 (Abcam) to determine immunoreactive area and the automated methods described earlier.

### 2.11 Statistics

All statistical analyses were conducted using GraphPad Prism 8. Data were first evaluated using one- or two-way analysis of variance (ANOVA) followed by Dunnett’s or Tukey’s multiple comparisons test to compare treatment groups to the control.

## 3 Results

### 3.1 CD38 is enriched in astrocytes in the human brain

To determine a detailed expression profile of CD38 across multiple human cell types and brain regions, we used the NETSseq sequencing platform (Stirparo et al., 2025; Xu et al., 2018) to investigate variations across brain regions of non-neurodegenerative disease control donors. NETSseq enabled deep transcriptomic analysis on a cell-type basis, allowing us to demonstrate that CD38 is preferentially expressed in astrocytes, with very low expression in other glial cell types and across 13 representative neurons from the cerebellum, midbrain, striatum, hippocampus, and cortex (Figure 2A). Within the astrocyte population, CD38 expression was higher in the forebrain and midbrain—regions relevant to PD pathology—with relatively low expression in the hindbrain, and in particular the astrocyte-like Bergmann glia of cerebellum (Figure 2B): one-way ANOVA analysis of CD38 expression across regions (F (7,267) = 30.47, p < 0.0001).

**FIGURE 2:**
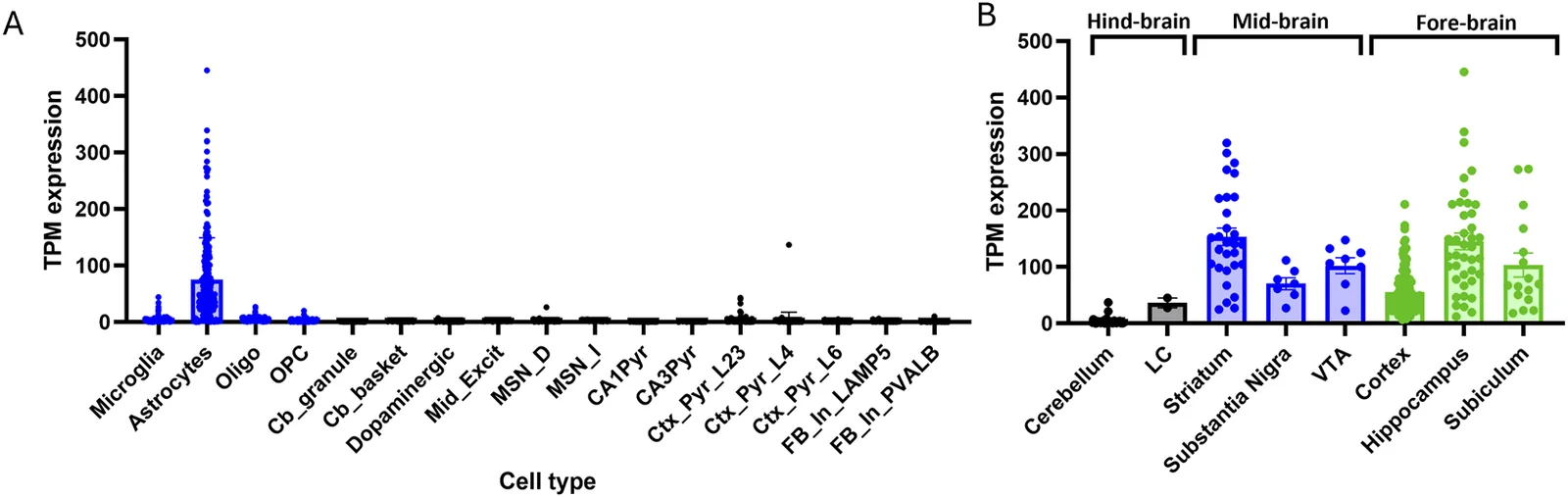
NETSseq CD38 expression in the human brain from control donors (n = 230 donors, n = 10–275 sorts per cell type). **(A)** CD38 mRNA expression in transcripts per million from nuclei isolated from control donor cell types: mature oligodendrocytes (Oligo), oligodendrocyte precursors cells (OPC), cerebellar granule cells (Cb_granule), cerebellar basket cells (Cb_basket), dopaminergic neurons from ventral tegmental area and substantia nigra (Dopaminergic), midbrain excitatory neurons (Mid_Excit), medium spiny neurons from direct and indirect pathway (MSN_D and MSN_I), CA1 hippocampal pyramidal neuron (CA1_Pyr), pyramidal neurons from layers 2 and 3 of various cortical regions (Ctx_Pyr_L23), pyramidal neurons from layer 4 of various cortical regions (Ctx_Pyr_L4), pyramidal neurons from layer 6 of various cortical regions (Ctx_Pyr_L6), LAMP5 positive interneurons from various cortical regions, CA1, dentate gyrus (FB_In_LAMP5), and parvalbumin positive interneurons from various cortical regions (FB_In_PVALB). Glial types in blue. **(B)** Astrocyte expression of CD38 in different brain regions. LC, locus coeruleus; VTA, ventral tegmental area. Graphs are presented as mean ± SEM (n = 2–130 sorts).

*In situ* hybridisation and immunohistochemistry demonstrated CD38 presence in the SN of both control and PD donors (Figure 3). Figures 3A,B illustrate control and PD SN sections, with the pink hybridisation signal representing CD38 RNA and brown neuromelanin identifying dopaminergic neurons, which are reduced in PD. Figure 3C shows an even distribution of CD38+ cells (orange/brown) alongside dopaminergic neurons (dark purple) in a control SN. In support of the NETSseq data, CD38 did not co-localise with the neuromelanin-containing dopaminergic neurons. Figure 3D highlights CD38 immunoreactivity in a PD donor SN, demonstrating the accumulation of CD38-expressing cells around surviving dopaminergic neurons and areas likely under stress in disease. Due to the limited availability of human tissue, these observations were not quantified and should therefore be considered qualitative. Validation in a larger cohort will be required to confirm these findings.

**FIGURE 3:**
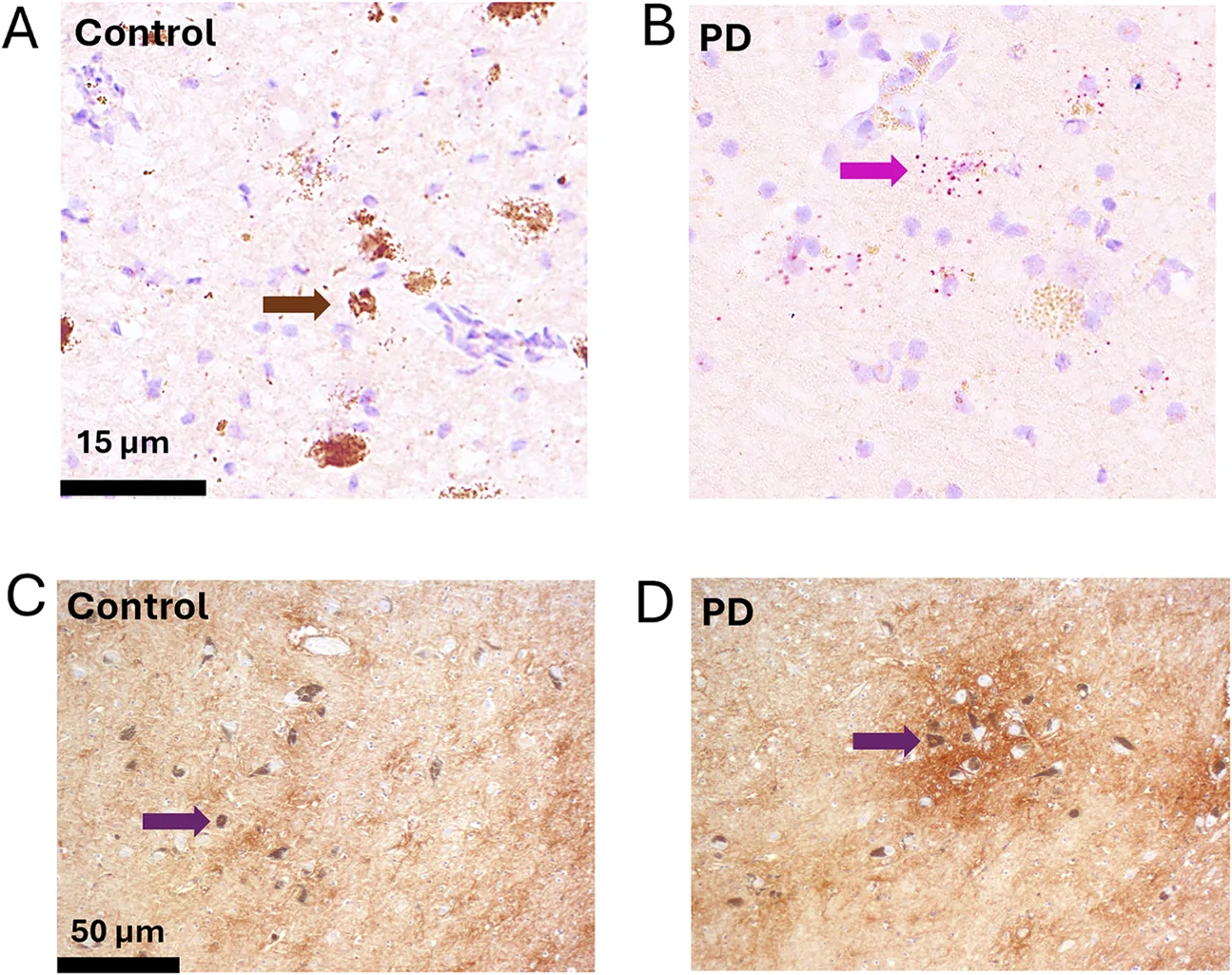
CD38 expression in human substantia nigra. *In situ* hybridisation (ISH) and immunohistochemistry (IHC) for CD38 was examined in control and PD donors. **(A,B)** are representative IHC images: haematoxylin for nuclei- purple, CD38 hybridisation signal- pink (and pink arrow), neuromelanin expressing dopaminergic neurons- brown (and brown arrow). Images obtained at 40x magnification. **(C,D)** are representative ICH images: orange/brown staining is CD38 immunoreactivity and dark purple shows the endogenous pigmentation of dopaminergic neurons (purple arrows). CD38 immunoreactivity was observed in PD SN around areas of surviving dopaminergic neurons **(D)**. Images obtained at 20x magnification.

### 3.2 CVN14 is a potent, selective, brain-penetrant CD38 inhibitor

CVN14 is a potent and selective inhibitor against human, rat, and mouse CD38, with IC_50_ values of 19.0, 5.1, and 2.5 nM, respectively (Figure 4, K_i_ = 9.50, 2.55, 1.25 nM respectively according to the Cheng–Prusoff equation). Selectivity has been tested against a range of other (human) NADases; CD157, PARP1, SIRT1, and NAMPT (Figure 4 and Supplementary Material of Stott et al., 2026). IC_50_ was >30,000 nM for each, representing >1,600× selectivity for CD38 over these enzymes.

**FIGURE 4:**
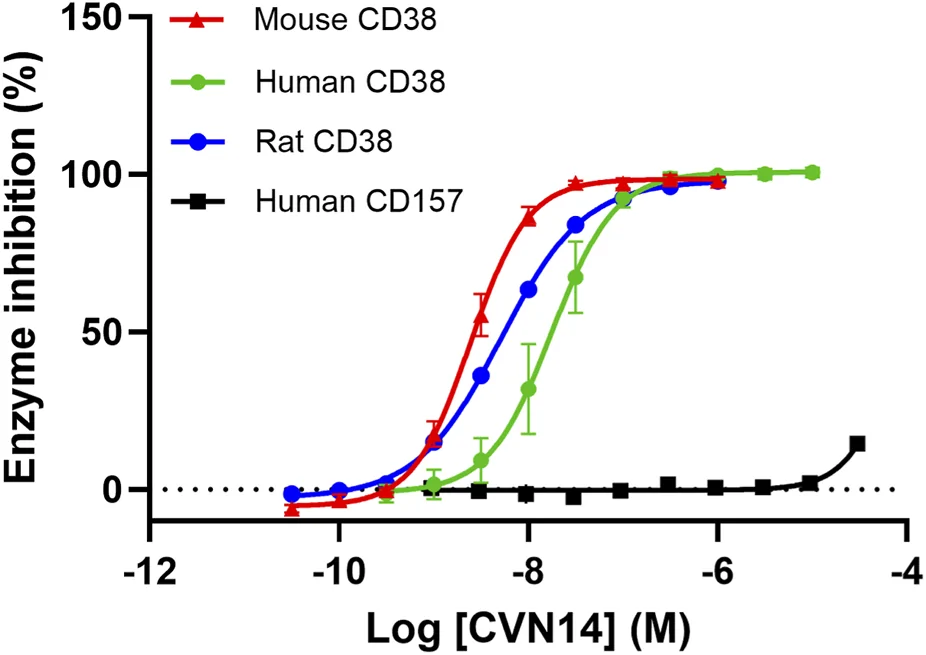
Enzyme activity in recombinant CD38/CD157 cell-free ε-NAD/NGD assays. Concentration–response relationships of CVN14 against human (19 nM, n = 16), rat (5.1 nM, n = 2), and mouse (2.5 nM, n = 4) CD38 and human CD157 (>30 μM, n = 2). Graphs presented as mean ± standard deviation.

To determine whether CVN14 is suitable for *in vivo* use in a rat model of PD, binding and PK properties were assessed, including CNS penetration. To understand the PK parameters of CVN14, rats were dosed with either 1 mg/kg *i*.*v*. (n = 3) or 3 mg/kg *p*.*o*. (n = 3). CVN14 showed excellent bioavailability (%F = 70), with a T_½_ of over 6 h and good exposure (Table 1), indicating suitability for once daily oral dosing in subsequent efficacy models. In a separate experiment to measure brain distribution at either 0.5 h (T_max_) or 8 h, rats were dosed *p*.*o*. with 10 mg/kg (n = 3/group) to reflect an upper proposed dose to be used in the *ex vivo* NAD^+^ and AAV1/2-hA53T-α-synuclein rat model experiments. CVN14 was found to be brain-penetrant with similar brain-to-plasma ratios at 0.5 h and 8 h post-dose, with fraction-unbound-in-brain and unbound-brain-to-plasma partition coefficients of 0.274 and 0.810, respectively (at 10 mg/kg 8 h post-dose).

**TABLE 1:** Rat PK properties of CVN14.

*p.o.* 3 mg/kg	Mean	Standard deviation
C_max_ (ng/mL)	12,696	835
T_max_ (h)	0.500	0
T_1/2_ (h)	6.16	1.29
T_last_ (h)	24.0	0
AUC_0–last_ (ng.h/mL)	119,499	7,546
*p.o.* 10 mg/kg		
Unbound brain		0.810
Unbound brain:plasma		0.274

C_max_, maximum concentration; T_max_, time at which C_max_ occurs; T_last_, time at last quantifiable concentration; AUC_0–last_, total exposure over observed sampling period.

### 3.3 Inhibition of CD38 raised brain NAD^+^

CVN14 increased rat brain NAD^+^ concentration in a dose-dependent manner from 1–10 mg/kg, with a ∼3-fold change from vehicle at 10 mg/kg (Figure 5A, one-way ANOVA: F (3,14) = 124.4, p < 0.0001). The mean vehicle and 1, 3, and 10 mg/kg [NAD^+^] were 27.27 ± 2.26, 36.39 ± 2.19, 57.75 ± 12.47, and 79.84 ± 2.42 μM, respectively (vehicle: n = 5, 1 mg/kg: n = 5, 3 mg/kg: n = 3, 10 mg/kg: n = −5). Brain concentrations of CVN14 increased linearly with dose: 0.27 μM ± 0.02 at 1 mg/kg, 0.9 ± 0.04 at 3 mg/kg, and 2.83 ± 0.35 at 10 mg/kg, measured 2 h post-dose.

**FIGURE 5:**
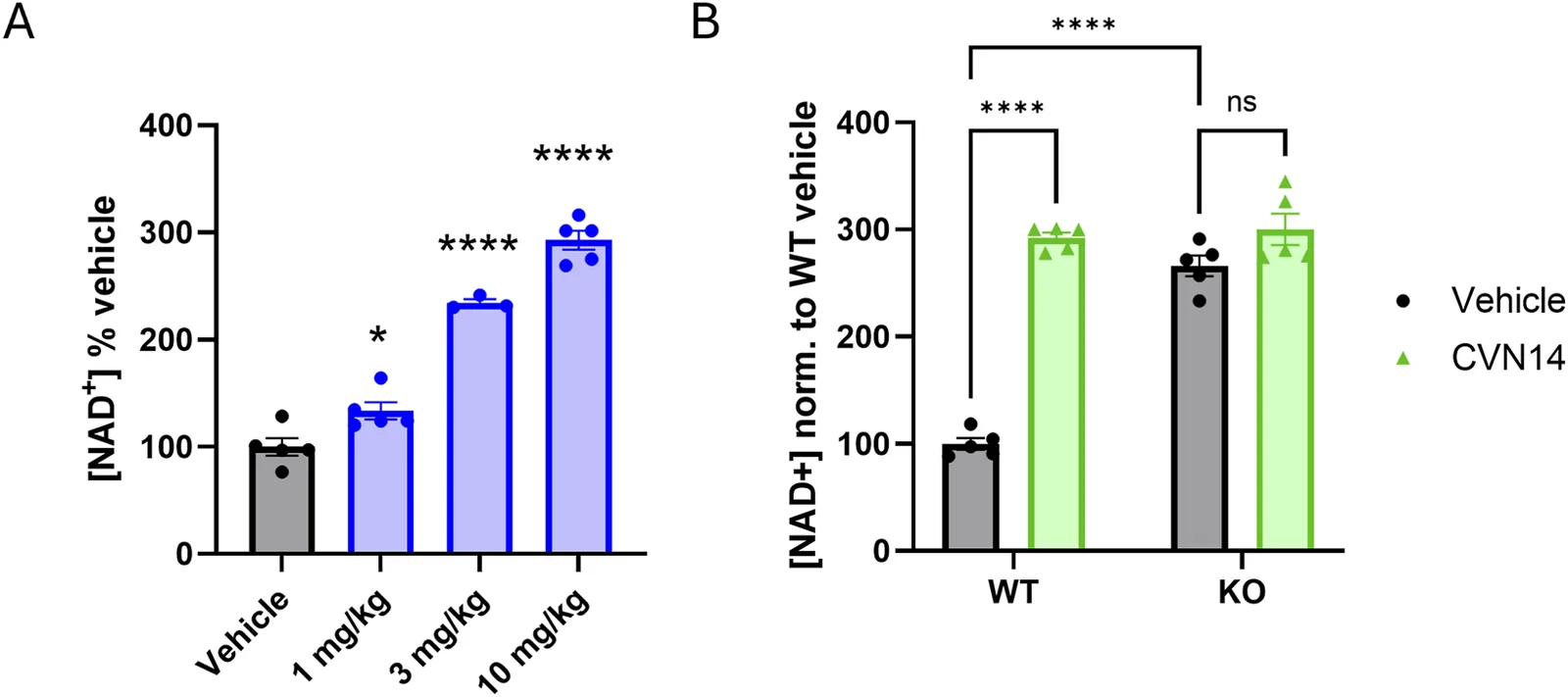
Rodent brain NAD^+^. **(A)** Rat brain NAD^+^ levels with increasing doses of CVN14, normalised to vehicle concentrations. One-way ANOVA with Dunnett’s multiple comparisons (*p = 0.024, ****p < 0.0001, vehicle: n = 5, 1 mg/kg: n = 5, 3 mg/kg: n = 3, 10 mg/kg: n = 5). **(B)** WT mouse and CD38 KO mouse brain NAD^+^ levels with vehicle and 10 mg/kg CVN14. Two-way ANOVA with Tukey’s multiple comparisons test (****p < 0.0001, n = 5 per group). Graphs presented as mean ± SEM.

To further demonstrate that CVN14 increases NAD^+^ levels via inhibition of CD38 and not another NADase, we conducted similar experiments in animals in which CD38 expression had been ablated. As CD38 KO rats are not readily available, we used CD38 KO mice. CD38 KO mice had a ∼3-fold increase in NAD^+^ compared to WT, similar to the effect of 10 mg/kg CVN14 in WT mice. CVN14 increased WT mouse brain NAD^+^ concentration, but no further increase (above that already observed with the genetic ablation) was seen in the KO (Figure 5B). WT mouse vehicle and 10 mg/kg [NAD^+^] were 30.3 ± 1.62 and 88.49 ± 1.49 µM, respectively. KO mouse vehicle and 10 mg/kg [NAD^+^] were 80.55 ± 3.00 and 90.90 ± 4.45 µM, respectively (n = 5 per group, two-way ANOVA: F (1,16) = 68.5, p < 0.0001, Tukey’s post hoc tests: WT vehicle vs. CVN14 vehicle p < 0.0001, KO vehicle vs. KO CVN14 p = 0.0932, WT vehicle vs. KO vehicle p < 0.0001). Plasma exposure with 10 mg/kg CVN14 was similar between WT and KO mice at 13.34 ± 1.40 and 13.53 ± 1.25 µM, respectively, at 2 h post-dose. These data demonstrate that the increase in NAD^+^ in WT rodents is likely due to inhibition of CD38 and not via action at other NAD^+^ modulating targets.

### 3.4 Inhibition of CD38 is associated with increased neuronal survival and reduced pSer129 α-synuclein levels *in vitro*

To understand how CD38 inhibition affects neuronal survival against a PD-relevant challenge, mixed primary cultures from rat ventral mesencephalon that included both dopaminergic neurons (TH immunoreactive) and astrocytes were exposed to vehicle or α-synuclein pre-formed fibrils and either vehicle, increasing doses of CVN14, or a positive control (BDNF or NMN: an NAD^+^ precursor). Under these conditions, α-synuclein–vehicle-exposed cultures showed a 55% reduction in TH+ neuron number compared to vehicle–vehicle (Figure 6A). CVN14 protected TH+ neurons from cell death caused by the α-synuclein challenge in a concentration-dependent manner, restoring cell numbers, on average, to 86.69% of control cultures at 300 nM (Figure 6A). Concurrently, the number of TH-expressing neurons that were positive for pSer129-α-synuclein increased by 132% at 300 nM following α-synuclein challenge (Figure 6B). CVN14 reduced this in a concentration-dependent manner, limiting the increase to 23.10% of control at 300 nM (Figure 6B). The data are from two independent cultures with six technical replicates per culture/condition and are presented as a percentage of unchallenged, vehicle-treated control cultures.

**FIGURE 6:**
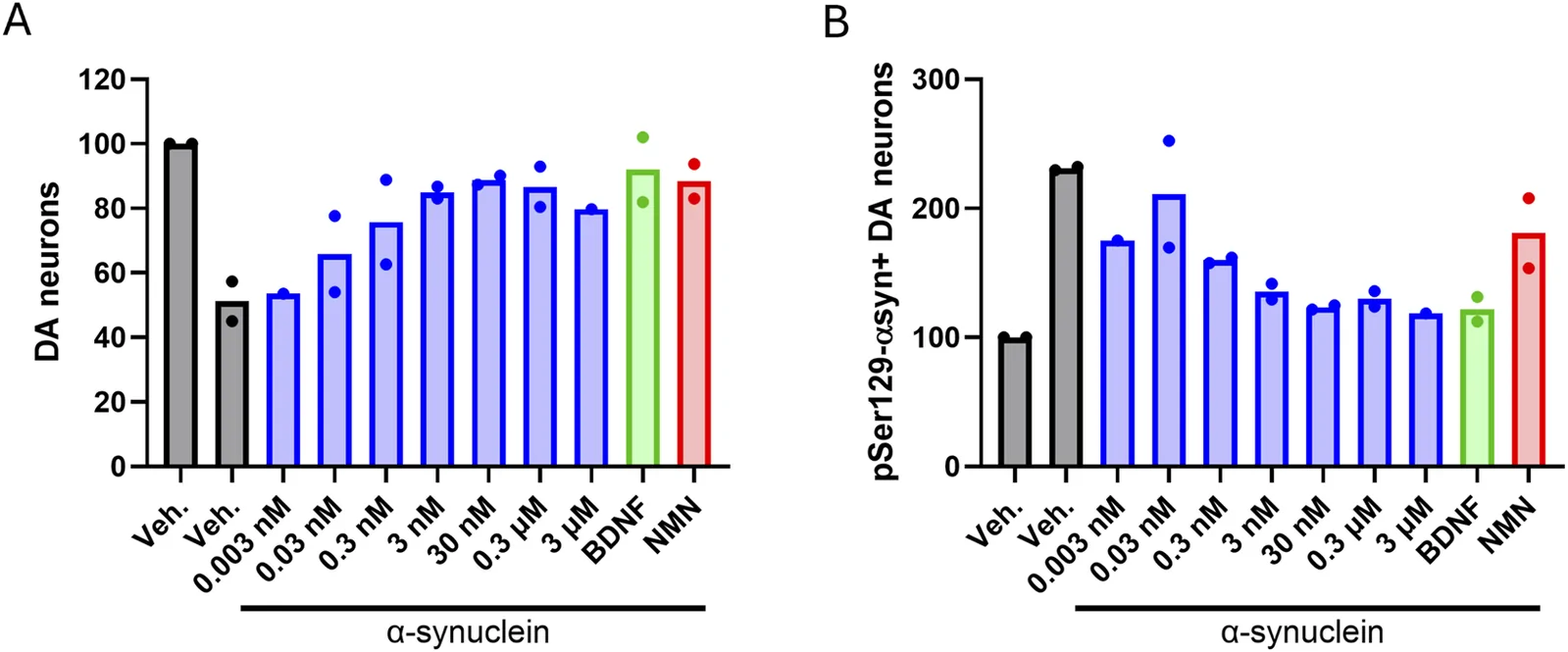
Neuroprotection and α-synuclein *in vitro.* **(A)** Quantification of the number of dopaminergic (TH-positive) neurons, with 500 nM α-synuclein challenge and CVN14, as well as positive controls BDNF (50 ng/mL) and NMN (5 mM). Presented as a percentage of unchallenged, vehicle-treated control cultures. **(B)** Quantification of the number of dopaminergic neurons that are positive for pSer129-α-synuclein as a percentage of unchallenged, vehicle-treated control cultures, with α-synuclein challenge and with increasing concentrations of CVN14, as well as positive controls BDNF (50 ng/mL) and NMN (5 mM). All data are from two independent cultures with six technical replicates per culture. Graphs presented as mean ± SEM.

### 3.5 Inhibition of CD38 improves motor function in an AAV1/2-hA53T-α-synuclein rat model of PD

To understand whether the observed decrease in α-synuclein by CVN14 treatment seen *in vitro* could be mimicked *ex vivo*, AAV1/2-hA53T α-synuclein or empty vector (AAV1/2-EV) was injected into the right SN of rats with the objective of measuring both motor impairment contralateral to the injection and levels of α-synuclein in the SN. Oral administration of vehicle or CVN14 began 1 day after surgery and continued for 42 days. Doses selected in this model were based on those shown to increase NAD^+^ levels in previous experiments. To measure the asymmetric motor function, the cylinder test was used to exploit the rats’ natural rearing behaviour when exploring novel environments via the placement of forepaws on the wall of the cylinder. As individual animals exhibited variation in baseline forepaw preference and exploratory behaviour, forepaw use at Day 42 was normalised to each animal’s baseline measurement. This within-animal analysis minimises the influence of pre-existing behavioural variability and allows treatment effects to be assessed relative to each animal’s own baseline. Rats injected with AAV1/2-EV placed both paws symmetrically when rearing, whereas rats injected with AAV1/2-hA53T α-synuclein preferentially used the unaffected, right (ipsilateral) forelimb at 42 days, indicating motor deficits on the contralateral side. CVN14 treatment restored this deficit in a dose-dependent manner, with dosed rats showing greater symmetry in forepaw use (Figure 7A). One-way ANOVA with Dunnett’s *post hoc* tests comparing all groups to the α-synuclein/vehicle showed an improvement in motor deficits with the 10 mg/kg dose of CVN14 (F (4,56) = 1.8, p = 0.0168; Dunnett’s test, EV/vehicle vs. α-synuclein/vehicle: p = 0.0109, α-synuclein/vehicle versus α-synuclein/10 mg/kg CVN14: p = 0.0274, vehicle: n = 12, α-syn/vehicle: n = 14, α-syn/0.3 mg/kg: n = 11, α-syn/1 mg/kg: n = 13, α-syn/10 mg/kg: n = 12). After termination, their brains were taken and processed to assess the amount of pSer129 α-synuclein in the SN. As expected, injection of AAV1/2-hA53T α-synuclein increased the detection of pSer129 α-synuclein in the SN compared to empty vector controls, confirming a well-established pathological phenotype in this model and in PD. SN pSer129 α-synuclein was quantified using immunohistochemical methods, measuring the immunoreactive area (Figure 7B). CVN14 treatment showed a mean numerical decrease in pathological α-synuclein with treatment that was not significantly different to the vehicle group (one-way ANOVA: F (4,53) = 2.45, p = 0.0517); Dunnett’s *post hoc* tests comparing α-synuclein/vehicle to α-synuclein/CVN14, p > 0.08 for each group, and vehicle/vehicle to α-synuclein/vehicle, p = 0.0153 (animal numbers-vehicle: n = 7, α-syn/vehicle: n = 10, α-syn/0.3 mg/kg: n = 11, α-syn/1 mg/kg: n = 11, α-syn/10 mg/kg: n = 8)) (Figure 7B).

**FIGURE 7:**
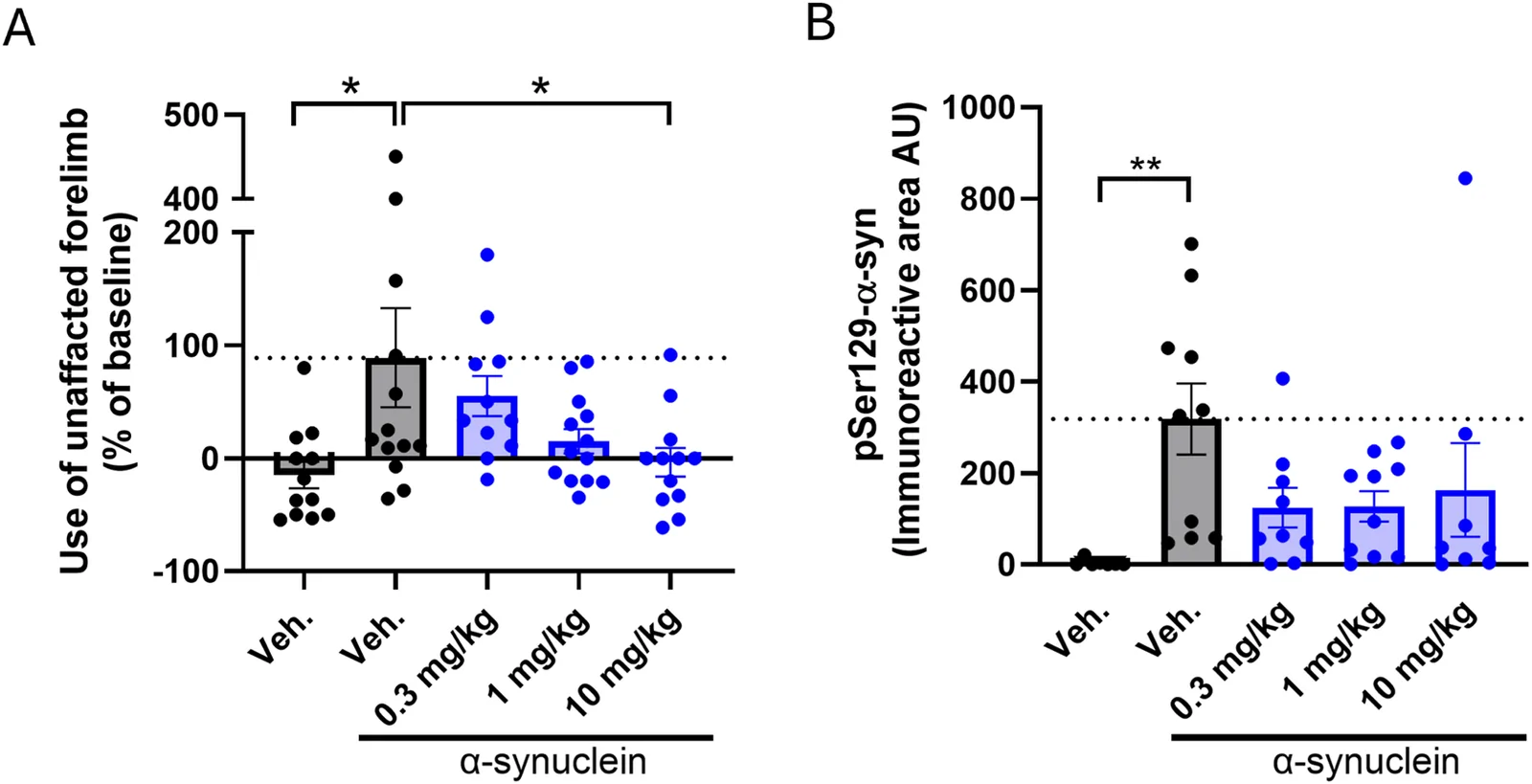
AAV1/2-hA53T-α-synuclein rat model of Parkinson’s disease. **(A)** Quantification of rat performance in the cylinder test, which measures forelimb asymmetry. Y-axis corresponds to % change in use of unaffected/ipsilateral paw from baseline (prior to surgery) to 42 days of once daily CVN14 dosing (*p < 0.05). Vehicle: n = 12, α-syn/vehicle: n = 14, α-syn/0.3 mg/kg: n = 11, α-syn/1 mg/kg: n = 13, α-syn/10 mg/kg: n = 12). **(B)** Quantification of substantia nigra pSer129 α-synuclein, detected immunohistochemically. The immunoreactive area was measured in arbitrary units (AU). Animal numbers-vehicle: n = 7, α-syn/vehicle: n = 10, α-syn/0.3 mg/kg: n = 11, α-syn/1 mg/kg: n = 11, α-syn/10 mg/kg: n = 8. One-way ANOVA with Dunnett’s multiple comparisons comparing all groups to α-syn/vehicle was performed for the quantifications in both **(A,B)**. Graphs presented as mean ± SEM.

## 4 Discussion

Our study provides the first pharmacological evidence that the selective inhibition of CD38 is beneficial in *in vitro* and *in vivo* models relevant to Parkinson’s disease, supporting further investigation of CD38 inhibition as a therapeutic strategy for the disease. It also supports the reported genetic links between CD38 and PD, as well as the disruption of NAD^+^ metabolism in PD pathophysiology.

Brain expression of CD38 has been observed in various mammalian species, including mice (Ceni et al., 2003), rats (Braidy et al., 2014; Yamada et al., 1997), and humans (Mizuguchi et al., 1995). Through NETSseq, our transcriptomic analyses showed a pronounced enrichment of CD38+ in human astrocytes, particularly within midbrain and forebrain regions which are highly relevant to PD pathology. Immunohistochemistry revealed an accumulation of CD38+ cells surrounding surviving SN neurons in PD, consistent with the recruitment of reactive astrocytes to areas of ongoing pathology and cellular stress. Given that CD38 is predominantly localised to the plasma membrane of astrocytes, with its catalytic site facing the extracellular space (Chini, 2009; Malavasi et al., 2008), CD38 is well-positioned to modulate NAD^+^ levels for utilisation by neurons and ultimately provide metabolic support and affording neuroprotective benefit.

Functionally, CD38 not only degrades NAD^+^ but also metabolises extracellular precursors such as NMN and nicotinamide riboside (NR) before they can enter intracellular salvage pathways (Yoshino, 2019). Accordingly, CD38 inhibition with CVN14 is expected to elevate both NAD^+^ and its precursors, including NMN and NR, in the extracellular space. Neurons may benefit from NAD^+^ elevation via connexin-mediated NAD^+^ transfer between astrocytes and neurons (Bruzzone et al., 2001; Condorelli et al., 2000; Kay et al., 2016) or through precursor uptake across the plasma membrane (Xie et al., 2020). Both mechanisms increase substrate availability and intracellular NAD^+^ synthesis through salvage pathways. While our *ex vivo* results confirmed increased bulk brain NAD^+^ after CD38 inhibition, distinguishing between intra- and extracellular pools and the specific cell types involved remains an important challenge. Additionally, the assay used in this study did not measure NADH, precluding an assessment of the NAD^+^/NADH ratio—an important indicator of cellular redox state and metabolic function.

Pharmacological inhibition of CD38 with the potent and selective inhibitor CVN14 elevated brain NAD^+^ *ex vivo.* Mechanistically, the restoration of NAD^+^ by CD38 inhibition triggers multiple downstream benefits, including reduced neuroinflammation, enhanced cellular metabolism, and improved clearance of misfolded proteins (Klinzing et al., 2024; Ocampo et al., 2013; Roboon et al., 2021). CVN14 showed evidence of increasing dopaminergic neuronal survival following α-synuclein challenge and reducing pathological α-synuclein *in vitro*. These findings require confirmation in a larger number of independent cultures. Although a numerical reduction in pathological α-synuclein was also observed *in vivo*, this did not reach statistical significance. Neuroprotection *in vivo* was not assessed in the present study. Future studies incorporating the stereological quantification of TH^+^ neurons will be required to determine whether CD38 inhibition protects nigral dopaminergic neurons. Given that α-synuclein is primarily degraded through lysosomal pathways in astrocytes, microglia, and neurons (Kim et al., 2013; H. J. Lee et al., 2008), it is plausible that elevated astrocytic NAD^+^ enhances phagocytic clearance of fibrils, while neuronal NAD^+^ may protect against α-synuclein aggregation by alleviating mitochondrial stress and inflammatory signalling (Yang et al., 2022). Consistent with observations of reduced NAD^+^ in PD brain and muscle (Mischley et al., 2023; Zhu et al., 2021), our findings highlight the therapeutic potential of restoring NAD^+^ levels, since CVN14 improved motor deficits in hA53T-α-synuclein rats.

Although CD38 is a key driver of age-associated NAD^+^ decline, and ageing is the greatest risk factor for PD, the present study was designed to evaluate the therapeutic potential of CD38 inhibition in disease-relevant models rather than to investigate ageing itself. Future studies in naturally aged animals, or aged PD models, will be important to determine how age-related changes in NAD^+^ metabolism influence the efficacy of CD38 inhibition and whether ageing further enhances the therapeutic benefit of this approach.

Other NAD^+^-boosting strategies have been explored in PD, including intravenous NAD^+^ (Gadol et al., 2019; Rutherford L. et al., 2024) and supplementation with NR or NMN (Covarrubias et al., 2021; Pérez et al., 2021). The NADPARK Phase 1 trial reported that high-dose NR was well-tolerated and raised brain NAD^+^, although clinical benefit was modest and variable (Brakedal et al., 2022). Given the limitations of precursor supplementation in the context of inefficient salvage pathways and hyperactive NADases in ageing or diseased cells (Conlon and Ford, 2022), our approach of enhancing NAD^+^ by reducing its consumption at the cellular astrocyte-neuronal level offers a potentially more targeted and durable strategy. By concentrating NAD^+^ increases at this level in those brain regions undergoing cellular stress and progressive neurodegeneration, CD38 inhibition may prove to be particularly effective in PD.

In summary, our study is the first pharmacological report demonstrating that the selective inhibition of CD38 is beneficial in both *in vitro* and *in vivo* PD models, including compelling evidence that the pharmacological inhibition of CD38 can improve motor deficits in a rat model of PD. Additional mechanistic studies will be required to fully elucidate the exact biochemical pathways mediating this CD38/NAD-induced protection. As CD38 inhibitors progress towards clinical evaluation, NAD^+^ and α-synuclein may serve as valuable biomarkers for monitoring target engagement and disease-modifying effects. Together, our findings underscore CD38 inhibition as a promising therapeutic approach for disease-modifying treatment in PD, thus justifying further investigation.

## Data Availability

The original contributions presented in the study are included in the article/Supplementary Material, further inquiries can be directed to the corresponding author.
