# Supplementary material for: CD38 inhibition increases neuronal survival and reduces α-synuclein in vitro, elevates brain NAD+, and reduces motor deficits in a Parkinson’s disease model
Source: Front Pharmacol. 2026 Oct 23;17:1927552. doi: 10.3389/fphar.2026.1927552 (PMC13739665; doi:10.3389/fphar.2026.1927552)
Supplement: Supplementary file 1 [file Supplementaryfile1.docx]

**Supplementary Table 1: Forelimb use during the cylinder test in rats injected with AAV1/2-hA53T α-synuclein and treated with vehicle or CVN14.** Forelimb paw contacts were recorded during the cylinder test at baseline (BL) and 42 days (D42) following AAV injection. Ipsilateral, contralateral, both-paw and total paw contacts are presented as mean ± SEM. Percentage asymmetry was calculated as [(ipsilateral−contralateral)/ipsilateral + contralateral + (both x 2)]×100, with positive values indicating preferential use of the ipsilateral forelimb. Groups comprised vehicle (Veh), vehicle + α-synuclein (Veh + α-syn), and α-synuclein-treated animals receiving CVN14 (0.3, 1 or 10 mg/kg).

|  |  | **Paw contacts** | | | | | | | |  |  |
| --- | --- | --- | --- | --- | --- | --- | --- | --- | --- | --- | --- |
|  |  | **Ipsilateral** | | **Contralateral** | | **Both** | | **Total** | | **% asymmetry** | |
| **Group (n)** | **Timepoint** | **Mean** | **SEM** | **Mean** | **SEM** | **Mean** | **SEM** | **Mean** | **SEM** | **Mean** | **SEM** |
| Veh | BL | 9.83 | 0.87 | 8.58 | 1.12 | 0.83 | 0.39 | 19.25 | 1.52 | 7.85 | 9.05 |
| (12) | D42 | 7.67 | 0.84 | 8.50 | 1.07 | 1.33 | 0.47 | 17.50 | 1.28 | -3.57 | 8.43 |
| Veh + α -syn | BL | 10.31 | 1.65 | 7.69 | 1.03 | 1.23 | 0.57 | 18.94 | 1.40 | 8.78 | 11.48 |
| (14) | D42 | 13.69 | 1.57 | 3.31 | 0.63 | 0.38 | 0.18 | 17.38 | 1.47 | 55.45 | 8.24 |
| 0.3 mg/kg + α-syn | BL | 9.92 | 1.37 | 9.00 | 1.19 | 1.08 | 0.38 | 20.00 | 1.57 | 7.01 | 10.19 |
| (11) | D42 | 13.00 | 1.65 | 3.00 | 0.59 | 0.67 | 0.22 | 18.00 | 1.51 | 61.28 | 6.28 |
| 1 mg/kg + α -syn | BL | 12.14 | 1.49 | 10.29 | 1.26 | 1.07 | 0.34 | 23.50 | 1.15 | 7.92 | 10.10 |
| (13) | D42 | 11.79 | 1.06 | 4.07 | 1.14 | 1.29 | 0.54 | 18.15 | 0.85 | 46.11 | 9.38 |
| 10 mg/kg + α -syn | BL | 12.83 | 1.25 | 9.58 | 1.08 | 0.75 | 0.30 | 23.17 | 1.53 | 15.07 | 7.81 |
| (12) | D42 | 11.75 | 1.48 | 4.83 | 1.27 | 1.25 | 0.46 | 17.83 | 1.98 | 39.17 | 11.98 |

**Supplementary Figure 1: CD38 antibody validation. (A)** Anti-CD38 antibody (Cell Signalling #51000) demonstrated clear membrane staining in a sub-population of macrophages in FFPE sections of spleen and tonsil. **(B)** Anti-CD38 (Cell Signalling #51000) produced compelling evidence for CD38 co-expression with GFAP.
